# Sortase A Mediated Bioconjugation of Common Epitopes Decreases Biofilm Formation in *Staphylococcus aureus*

**DOI:** 10.3389/fmicb.2020.01702

**Published:** 2020-07-30

**Authors:** Poonam Kumari, Yutika Nath, Upadhyayula Surayanarayana Murty, Velayutham Ravichandiran, Utpal Mohan

**Affiliations:** ^1^Department of Biotechnology, National Institute of Pharmaceutical Education and Research, Guwahati, India; ^2^Department of Medicinal Chemistry, National Institute of Pharmaceutical Education and Research, Kolkata, India

**Keywords:** Sortase A, biofilm, LPXTG, *S. aureus*, infection

## Abstract

*Staphylococcus aureus* is one of the most notorious pathogens and is frequently associated with nosocomial infections imposing serious risk to immune-compromised patients. This is in part due to its ability to colonize at the surface of indwelling medical devices and biofilm formation. Combating the biofilm formation with antibiotics has its own challenges like higher values of minimum inhibitory concentrations. Here, we describe a new approach to target biofilm formation by Gram positive bacteria. Sortase A is a transpeptidase enzyme which is responsible for tagging of around ∼22 cell surface proteins onto the outer surface. These proteins play a major role in the bacterial virulence. Sortase A recognizes its substrate through LPXTG motif. Here, we use this approach to install the synthetic peptide substrates on*S. aureus.* Sortase A substrate mimic, 6His-LPETG peptide was synthesized using solid phase peptide chemistry. Incorporation of the peptide on the cell surface was measured using ELISA. Effect of peptide incubation on *Staphylococcus aureus* biofilm was also studied. 71.1% biofilm inhibition was observed with 100 μM peptide while on silicon coated rubber latex catheter, 45.82% inhibition was observed. The present work demonstrates the inability of surface modified *S. aureus* to establish biofilm formation thereby presenting a novel method for attenuating its virulence.

## Introduction

Most bacteria colonize on surfaces to form three-dimensional clusters called Biofilms. Bacterial populations associated to biofilms generally create a penetration barrier to antibiotics (Xu et al., 2000; Davies, 2003). Biofilm formation is one of the most important virulence mechanisms of many bacterial pathogens and thus considered to be the major cause of nosocomial infections especially in post-surgical and immune-compromised patients (Mirian et al., 2013; Zinaida et al., 2019). Biofilm can grow on both natural and artificial surfaces and guard the bacteria against the antibiotic therapy as well from host immune system (Arciola et al., 2018).

*Staphylococcus aureus* is the most common pathogen, which is involved in nosocomial infections and has been associated with significant mortality among hospitalized patients (Von-Eiff et al., 2001; Mulcahy and McLoughlin, 2016; Khatoon et al., 2018). The ability of *S. aureus* to form multilayered adherent biofilm to the surface of indwelling medical devices including catheters and medical implants, expressing series of toxins make them tolerant toward host defense mechanisms and common antibiotics (Neopane et al., 2018).

Sortase A is one of the most important enzymes present on the cell surface of Gram positive bacteria including *S. aureus*. In 1999, Schneewind and colleagues discovered that Sortase A recognizes the LPXTG motif present at the C-terminus of cell surface proteins and recruits them to the peptidoglycan cell wall building block, lipid II (Mazmanian et al., 1999). The search for molecules that can inhibit Sortase A is one of the promising approaches for the development of innovative strategies to attenuate bacterial virulence (Cascioferro et al., 2015; Bi et al., 2016; Zhang et al., 2016) and biofilm formation (Parrino et al., 2019). Wang et al. (2018) demonstrated that an oligopeptide LPRDA can be a potential anti-infective strategy for the treatment of *S. aureus* infections. They developed an effective inhibitor of Sortase A based on LPXTG substrate sequence and used it against *S. aureus-*induced mastitis in a mouse model (Wang et al., 2018). Naturally occurring peptides were also used against the Sortase A and Sortase B of the Gram positive bacteria. The group reported Conus venom peptides that inhibit the activity of Sortases (Sameera and Rashid, 2019).

In 2010, *S. aureus* surface was re-engineered using its own Sortase A with fluorescein and biotin (Nelson et al., 2010) for the first time. This study proved that the native Sortase A can also recognize and couple synthetic molecules containing LPXTG motif onto the peptidoglycan layer of Gram positive bacteria. In 2014, Veeman’s group studied the time-dependent incorporation of synthetic substrate on *S. aureus* cell wall. The group found that the maximum incorporation occurred at the stationary phase and it was not affecting the expression of native Sortase A substrate which is clumping factor, A/B and protein A (Man̆ásková et al., 2014). In 2016, the same group studied the effect of substrate alterations and concluded that sorting was more if positively charged amino acid was present after the recognition motif and also when the LPMTG was present instead of LPETG (Man̆ásková et al., 2016). The previous studies have not studied the effect of peptide incorporation on the ability of biofilm formation of *S. aureus* and their ability to attenuate the pathogen’s virulence was not evaluated.

In the present study, we have synthesized a novel 6His-LPETG peptide and incorporated it on the cell wall of *S. aureus.* We further evaluated the ability of this peptide to inhibit biofilm formation by Gram positive bacteria. We also analyzed the level of biofilm inhibition on catheter mediated by the peptide. We present here an innovative approach to attenuate the virulence associated with actively colonizing bacteria of biofilm which can be developed into alternative strategies to combat antibiotic resistant Gram positive populations related to nosocomial infections.

## Materials and Methods

### Chemicals, Bacterial Strains and Media Used

Trifluoroacetic acid (TFA), piperidine, thioanisole, 1,2- Ethanediol, diethylether, N,N′-dimethylformamide (DMF), Diisopropylethyl amine (DIPEA), rink amide resins, 1-Hydroxybenzotriazole (HOBt), and O-(benzotriazol-1-yl)-N,N,N′,N′-tetramethyluronium hexafluorophosphate (HBTU) were obtained from Sigma Aldrich, N-α-Fmoc protected amino acids were obtained from Novabiochem (Merck, United States) and bacterial media was obtained from Himedia^TM^ (India). 6X-Histidine and FLAG-LPETG peptide were purchased from Genscript Biotech (United States). All antibodies for ELISA and Confocal microscopy were purchased from Invitrogen. ProLong Gold antifade was purchased from Thermo Fisher Scientific. The experimental bacterial strains, *Staphylococcus aureus* (MTCC 3160), *Enterococcus faecalis* (MTCC 3159) and *Escherichia coli* (MTCC 42) were procured from Microbial Type Culture Collection (IMTECH, Chandigarh, India).

#### Peptide Synthesis and Characterization

Sortase A peptide substrate (HHHHHHLPETG) was synthesized *via* solid-phase peptide synthesis using 9-flurorenyl-methoxycarbonyl (fmoc)-chemistry (Coin et al., 2007; Vergel et al., 2014; Méndez et al., 2017). Rink amide resin was soaked overnight in dimethylformamide (DMF). For all the subsequent coupling reactions, Fmoc protected amino acids were activated by O-benzotriazole-N,N,N′,N′-tetramethyl-uronium-hexafluoro-phosphate (HBTU), hydroxybenzotriazole (HOBt) and N,N-Diisopropylethylamine (DIPEA) with resin in DMF and shaken at room temperature for 4 h. Deprotection of Fmoc-protecting group was done in 20% piperidine in DMF for 20 min.

The peptide cleavage reaction was done using m-cresol, thioanisole, 1,2-ethanedithiol (EDT) and trifluoroacetic acid in 2:2:1:20 ratio for 12 h at room temperature. Cleaved peptide was then filtered through glass wool in ice cold diethylether, pelleted, resuspended 6-7 times in diethylether and dried. The peptide was purified using Reversed Phase High Pressure Liquid Chromatography (RP-HPLC) (Dionex Ultimate 3000, Thermo Scientific) and absorbance measurements were made at 210 nm (León-Calvijo et al., 2015). A hypersil gold C-18 column was used for the purification of the peptide using an acetonitrile: water gradient containing 0.1% TFA. The purified peptide was further verified using mass spectrometry (MALDI) (AUTOFLEX SPEED, Bruker).

### Conjugation of 6His-LPETG Peptide on Bacteria

Overnight culture of bacteria was diluted to 1/100 in tryptic soy broth and treated with varying concentrations of desired peptide for 24 h at 37°C (Nelson et al., 2010; Man̆ásková et al., 2016). After 24 h, cells were pelleted and washed with 1XPBS and stored at 4°C till further use on the same day.

### Evaluating Peptide Conjugation Using ELISA

6His-LPTEG labeled *S. aureus* cells were fixed in 4% paraformaldehyde for 1 h. Cells were washed with 1X PBS and incubated with rabbit Anti-6His primary antibody overnight at 4°C. Cells were washed with 1X PBS and incubated with goat anti-rabbit HRP (Horseradish Peroxidase) conjugated secondary antibody for 2 h at room temperature and again washed three times with 1X PBS. TMB (3,3′,5,5′-Tetramethylbenzidine) Substrate was added into each well, incubated in the dark for 30 min and absorbance was measured at 450 nm (Ohsawa et al., 2015).

The same experiment was repeated for *E. faecalis* using mouse anti-6His primary antibody and goat anti-mouse AP (Alkaline Phosphatase) conjugated secondary antibody. pnPP (*p*-Nitrophenyl Phosphate) substrate was added for 20 min. After 20 min stop solution was added and absorbance was measured at 405 nm.

### Confocal Microscopy

Overnight culture of *S. aureus* was diluted to 1/100 in tryptic soy broth and treated with varying concentrations of our desired peptide for 24 h at 37°C. After 24 h, cells were harvested and washed with 1X PBS. Cells were fixed in 4% paraformaldehyde for 1 h and then incubated with Alexa Fluor IgG secondary antibody for 1.5 h. Cells were washed once with 1X PBS and 40 μl of cell suspension was mounted on glass slides with 10 μl of ProLong Antifade mounting media. The slides were incubated at 4°C overnight before acquiring images. Cells were imaged by Leica Microsystems confocal microscope (TCS SP8).

### Crystal Violet Biofilm Formation Assay

Bacteria were grown overnight in tryptic soy broth and normalized to 0.01OD_600_. Diluted bacterial suspension was dispensed in sterile 96-well polystyrene plate containing varying concentrations of the desired peptide. The 96-well plate was incubated statically at 37°C for 24 h. Wells were washed three times with 1XPBS and air-dried. Adherent cells were stained with 0.1% (w/v) crystal violet and *A*_570_ was measured (O’Toole, 2011; Shukla and Rao, 2017).

Percentage of Biofilm formation was calculated using the formula: (test_OD_ /negative control_OD_) × 100. Percentage inhibition was calculated using the formula: (negative control_OD_ - test_OD_ / negative control_OD_) × 100 where negative control is the sample without peptide treatment.

### Scanning Electron Microscopy

*S. aureus* was grown overnight in tryptic soy broth and normalized to 0.01 OD_600_. Diluted bacteria was dispensed onto a coverslip in a sterile 96-well polystyrene plate containing varying concentrations of the desired peptide. The plate was incubated statically at 37°C for 24 h. Wells was washed three times with 1XPBS. The biofilm was fixed with 4% formaldehyde at 4°C for 12 h. Subsequently the biofilm was washed twice with 1XPBS and dehydrated through a series of increasing concentration of graded alcohol series (Fratesi et al., 2004; Harriott and Noverr, 2009; Asahi et al., 2015). The samples were visualized using Field Emission Scanning Electron Microscope (Sigma, Zeiss).

### Biofilm Formation Assay on Catheter

Silicon coated rubber latex catheter was used for the *S. aureus* biofilm formation assay. Circular disk (1.88 cm^2^) of catheter was excised and placed in 96-well plate. *S. aureus* (0.01 OD_570_) with our desired peptide was incubated with catheter for 24 h at 37°C. After 24 h, crystal violet assay was used to quantify biofilm formation by *S. aureus* (Djeribi et al., 2012; Colomer-Winter et al., 2019).

### Statistical Analysis

All graphs were constructed using GraphPad PRISM 8.0. Comparisons among groups were done using one-way ANOVA. *P* < 0.05 was considered to be statistically significant.

## Results

### Synthetic Peptide Design and Surface Recruitment on *S. aureus*

In this present work, we have designed 6His-LPETG peptide based on the Sortase A mechanism of action. LPETG is the conserved recognition motif near the C-terminus of the cell surface proteins of *S. aureus* (Maresso and Schneewind, 2008). We envisaged that 6-His epitope along with LPETG motif will allow us to graft the surface of the bacteria with epitopes which can further be utilized to target the bacteria using 6-His antibodies.

**FIGURE 1:**
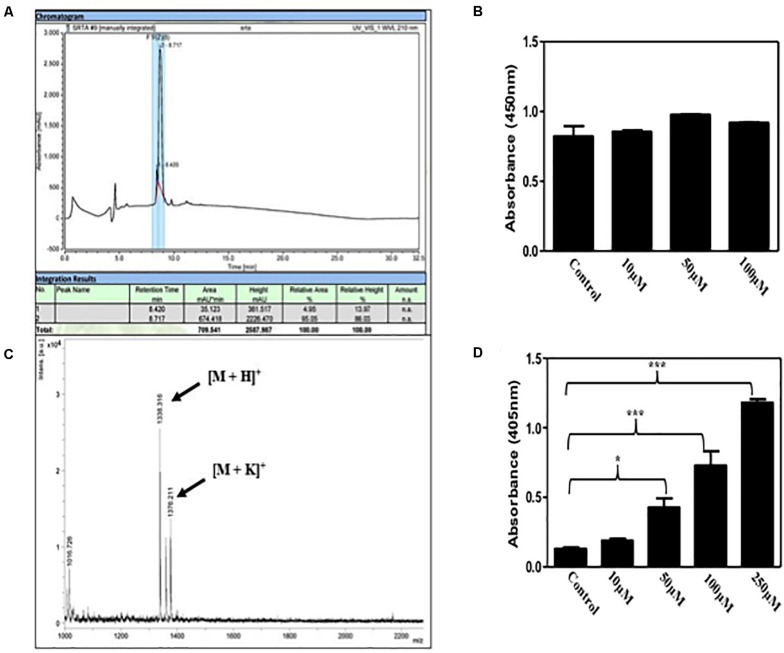
**(A)** Reversed phase HPLC chromatogram of the 6His-LPETG peptide with retention time 8.717 min. **(B)** MALDI-TOF analysis peaks of 6His-LPETG peptide. 6His-LPETG peptide conjugation on cell surface of **(C)** *S. aureus* and **(D)** *E. faecalis* as measured by ELISA done in triplicates. *P*< 0.05 was considered as significant. Error bars are mean ± SEM. *p < 0.05, ***p < 0.001.

The peptide was synthesized using Fmoc solid phase peptide chemistry (Stawikowski and Fields, 2012). It has been shown that LPETG C-terminus interferes with Sortase A activity (Popp et al., 2009; Nelson et al., 2010). Therefore, in our synthesis procedure rink amide resin was used to add amide group at the C-terminus of the peptide. After reversed phase HPLC, fractions were mixed with alpha-Cyano-4-hydroxycinnamic acid and analyzed by MALDI-TOF mass spectrophotometer. Mass spectra of the purified peptide exhibited strong peak enrichment at 1338.351 and 1376.21 amu (Figures 1A,B) and masses corresponds to [M+H]^+^ and [M+K]^+^ ions, respectively, for 6His-LPETG peptide.

It has been reported that natural occurring Sortase A exhibits selectivity to recognize LPETG motif (Mao et al., 2004). Incorporation of our desired peptide on bacterial cells was confirmed by ELISA. We incubated *S. aureus* with varying concentrations of peptide; 10, 50, and 100 μM but we did not observe any significant difference in color change (Figure 1C) which has been corroborated by the reports that suggest the binding of protein A and protein G to the Fc portion of IgG molecules (Falugi et al., 2013; Kobayashi and DeLeo, 2013; Choe et al., 2016). Since protein A and G are absent on the cell surface of *E. faecalis*, we performed the same experiment to see the visible color change with *E. faecalis*. Our results provide evidence for the incorporation of our synthetic peptide on the bacterial surface via Sortase A (Figure 1D). In addition, we analyzed the affect of 6His-LPETG peptide incubation on *E. coli*. There was no visible change on the *E. coli* cell surface as exhibited by confocal images (Supplementary Figure S1). *S. aureus* proteins bind to the Fc region of IgG molecules and manipulate the host immune response (Atkins et al., 2008; Thammavongsa et al., 2015; De Jong et al., 2019). Further, *S. aureus* Fc binding property was visualized using confocal microscopy. We observed that the decrease in fluorescence at 100 μM 6His-LPETG peptide (Supplementary Figure S2) indicated reduced binding of IgG on cell surface post 6His-LPTEG recruitment.

### Surface Recruited LPETG Synthetic Peptide Inhibits Biofilm Formation

**FIGURE 2:**
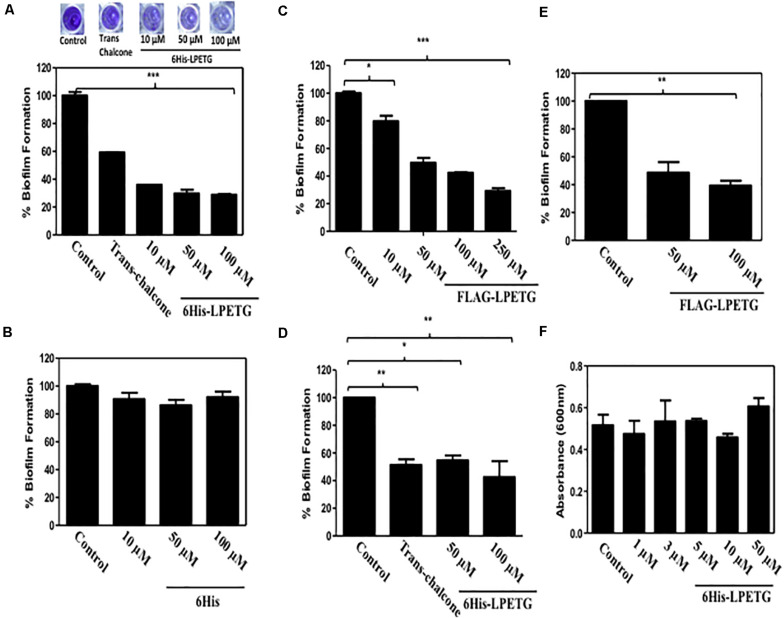
0.1% Crystal Violet assay with *S. aureus* using **(A)** 6His-LPETG, **(B)** 6His, and **(C)** FLAG-LPETG at indicated concentrations. One hundred micro meter Trans-chalcone was used as positive control in crystal violet assay. Peptide was incubated with 1/100 diluted *S. aureus* cell culture at indicated concentrations and absorbance (OD_600_) was measured after 24 h. 0.1% Crystal Violet assay with *E. faecalis* using **(D)** 6His-LPETG and **(E)** FLAG-LPETG at indicated concentrations. **(F)** *S. aureus* cell viability assay in the presence of 6His-LPETG. All the experiments weredone is triplicates and *P*< 0.05 was considered as significant. Error bars are mean ± SEM. *p < 0.05, **P < 0.01, ***p < 0.001.

Next we wanted to evaluate the activity of our synthetic peptide for biofilm inhibition. Synthetic Sortase A substrate peptide was incubated with the *S. aureus* with concentration upto 100 μM. We also used trans-chalcone as positive control as it is a known inhibitor of Sortase A (Wallock-Richards et al., 2015; Zhang et al., 2017). The crystal violet assay was performed to visualize the inhibition of biofilm formation in the presence of our synthetic peptide. Our results clearly demonstrated that the 6His-LPTEG peptide has immense potential to reduce biofilm formation (Figure 2A). One hundred micro molar of 6His-LPETG in *S. aureus* exhibited 71.1% biofilm inhibition while trans-chalcone at the same concentration was showing 40.77% inhibition. The negative control 6His peptide sans LPETG motif had no effect on biofilm reduction (Figure 2B). To further validate our study, FLAG-LPETG peptide was used in the biofilm inhibition assay. FLAG-LPETG was also showing anti-biofilm activity (Figure 2C) which confirms that exogenous Sortase A substrates may be used as anti-biofilm agents.

To eliminate the possibility that our peptide might be anti-microbial, we grew the bacterial cells in the presence of 6His-LPETG peptide. Our results indicated that the peptide has no visible effect on the viability of *S. aureus* (Figure 2F). We also wanted to investigate whether other bacteria expressing surface Sortase A engaged in biofilm formation can be inhibited by our peptide. *Enterococcus faecalis* is another Gram positive bacteria expressing cell surface Sortase A enzyme. *E. faecalis* was incubated with 50 and 100 μM of either 6His-LPETG or FLAG-LPETG. The results clearly indicated that both peptides have biofilm inhibitory activity against the bacteria (Figures 2D,E).

**FIGURE 3:**
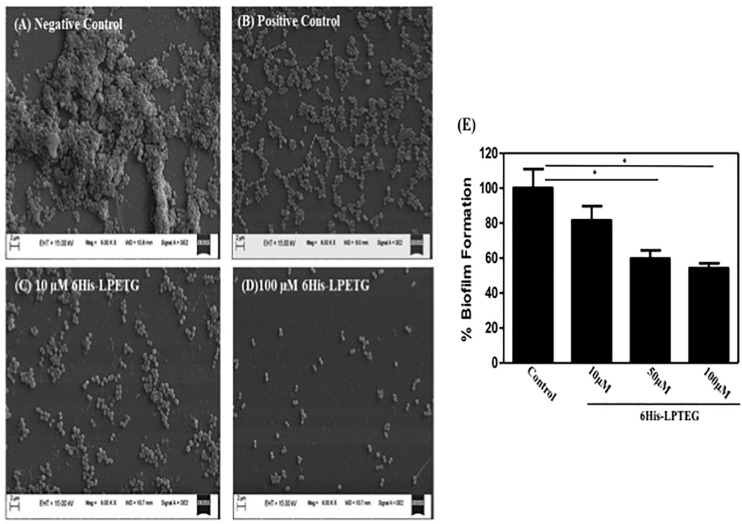
Representative SEM image of *S. aureus* biofilm formation using different concentrations of synthetic peptide. Images were acquired at 6000X magnification, **(A)** Negative Control **(B)** 100 μM Transchalcone used as positive control, 6His-LPETG peptide at **(C)** 10 μM and **(D)** 100 μM. **(E)** Biofilm formation by *S. aureus* as assayed by crystal violet, growing on silicone coated latex catheter disks in the presence of 6His-LPETG. Experiment was done is triplicates and *P* < 0.05 was considered as significant. Error bars are mean ± SEM. *p < 0.05.

To further confirm the activity of our peptide, we used field emission scanning electron microscope to demonstrate the anti-biofilm effect of 6His-LPETG. Representative SEM images clearly showed that the peptide, at the concentration of 10 μM, has an anti-biofilm activity against *S. aureus* (Figures 3A–D). The biofilm inhibition escalated as we increased the concentration of our 6His-LPETG peptide to 100 μM. The SEM images corroborated our biofilm inhibition hypothesis.

We also wanted to know whether the 6His-LPETG peptide has a standalone anti-biofilm activity. We used Design Peptide Against Bacterial Biofilms database (dPABBs) database (Sharma et al., 2016) to predict its possible anti-biofilm activity. dPABB is a web server that predicts the anti-biofilm activity of peptides and proteins based on it’s amino acid composition and positional preferences. The output score of −0.34 for 6His-LPETG and −1.26 for FLAG-LPETG (Supplementary Figures S3, S4) suggested that both the peptides did not have standalone anti-biofilm activity but their recruitment on Gram-positive bacteria exhibits biofilm inhibition.

Major proteins involved in biofilm formation like Protein G (SasG), Serine aspartate repeat protein (SrdC), SasX, Clumping factor B (ClfB), Serine-rich adhesin for platelets (SraP) (Corrigan et al., 2007; Speziale et al., 2014) are the substrates for Sortase A and have a conserved LPXTG motif at their C-terminus (Supplementary Figure S5). As our synthetic peptide also has LPETG motif, so it may compete with the cell surface proteins of the *S. aureus* involved in the biofilm formation thus inhibiting the biofilm formation which require further validation.

### The 6His-LPTEG Peptide Inhibits Biofilm Formation of *S. aureus* on Catheter

*S. aureus* is one of the major pathogens associated with infections caused by medical devices including catheter associated urinary tract infections (CAUTI) in hospitalized patients (Xu et al., 2020). *S. aureus* and *S. epidermidis* are estimated to cause around 87% bloodstream, 40–50% prosthetic heart valve infection and 50–70% catheter biofilm infections (Chen et al., 2013). Immense efforts has been dedicated to develop anti-biofilm coating on catheter and other medical devices to circumvent associated infections and post surgical complications in hospitalized patients (Zhu et al., 2019; Von Borowski et al., 2020; Xie et al., 2020).

We used a silicon coated rubber latex catheter and incubated it with *S. aureus* and our peptide. Crystal violet assay was utilized to visualize *S. aureus* biofilm formation on catheter. Our results demonstrate that 6His-LPETG peptide used in our studies is able to reduce the biofilm formation significantly on the surface of catheter (Figure 3E). One hundred micro molar of 6His-LPETG peptide was able to inhibit the biofilm formation for upto 45.82%.

## Discussion

*S. aureus* is one of the key pathogens associated with hospital acquired infections involving medical devices including catheters, pacemakers, contact lenses, and dentures (Lewis, 2001; Piozzi et al., 2004). Also, *S. aureus* expresses a range of virulence factors helping it to escape host defense and making it resistant to common antibiotics. Since *S. aureus* biofilms on natural and artificial surfaces imposes serious threat to both hospitalized and immune-compromised patients, potent strategies to inhibit or eradicate its spread would be very instrumental in managing *S. aureus* infection and pathogenesis. We have synthesized and evaluated a peptide substrate for cell surface enzyme Sortase A which exhibits significant anti-biofilm activity. As this peptide has an LPETG motif, it could further be tested against other Gram positive bacteria having Sortase A enzyme. The present strategy of using synthetic Sortase substrate(s) as anti-biofilm agents has immense potential in developing molecules, which can attenuate the virulence of Gram positive bacteria. We also see an opportunity of developing and testing similar LPETG motif containing peptides for inhibiting biofilm formation in other Gram positive bacteria. Further studies are being pursued by our laboratory to explore the ability of such peptides to inhibit protein G and protein A recruitment on the bacterial cell surface.

Studies and observations have demonstrated that higher concentration of antibiotics used to eradicate biofilm bacteria further enhances their resistance against conventional antibiotics and development of multi drug resistant strains (Hoyle and Costerton, 1991; Moreau-Marquis et al., 2008; Parrino et al., 2019). The ability of pathogenic biofilm to sustain antibiotics leads to treatment failure and recurrence of infection in any hospital setting. Widespread use of medical devices and implants favors colonization of pathogenic bacteria leading to infection which can be detrimental in immunocompromised post surgical patients. There is always a need to design efficient strategies to combat pathogenic biofilm to eradicate associated infections in medical implants and other devices.

The advantage of our strategy is that it is neither altering any biological process within the bacteria nor inhibiting it. Thus, it is not putting any selective pressure on bacterial population which is one of the major concerns of antibiotic therapy. Our peptide is using bacterial machinery to recruit itself on the cell surface and then hinder the process of biofilm formation.

Our strategy can potentially be developed and utilized to make anti-biofilm surfaces for clinical applications. We have grafted the cell surface of *S. aureus*, a difficult pathogen, with a very common epitope. Grafting of common epitopes on the bacterial cell surfaces can help us to further investigate the ability of host immune system to target this bacteria.

## Data Availability Statement

The raw data supporting the conclusions of this article will be made available by the authors, without undue reservation, to any qualified researcher.

## Author Contributions

UM and PK designed the present work. PK and YN performed the experiments. VR, USM, and UM wrote the manuscript. All authors contributed to the article and approved the submitted version.

## Conflict of Interest

The authors declare that the research was conducted in the absence of any commercial or financial relationships that could be construed as a potential conflict of interest.
