# Supplementary material for: Sortase A Mediated Bioconjugation of Common Epitopes Decreases Biofilm Formation in Staphylococcus aureus
Source: Front Microbiol. 2020 Jul 30;11:1702. doi: 10.3389/fmicb.2020.01702 (PMC7438799; doi:10.3389/fmicb.2020.01702)
Supplement: Supplementary file 1 [file Data_Sheet_1.PDF]

Supplementary Material

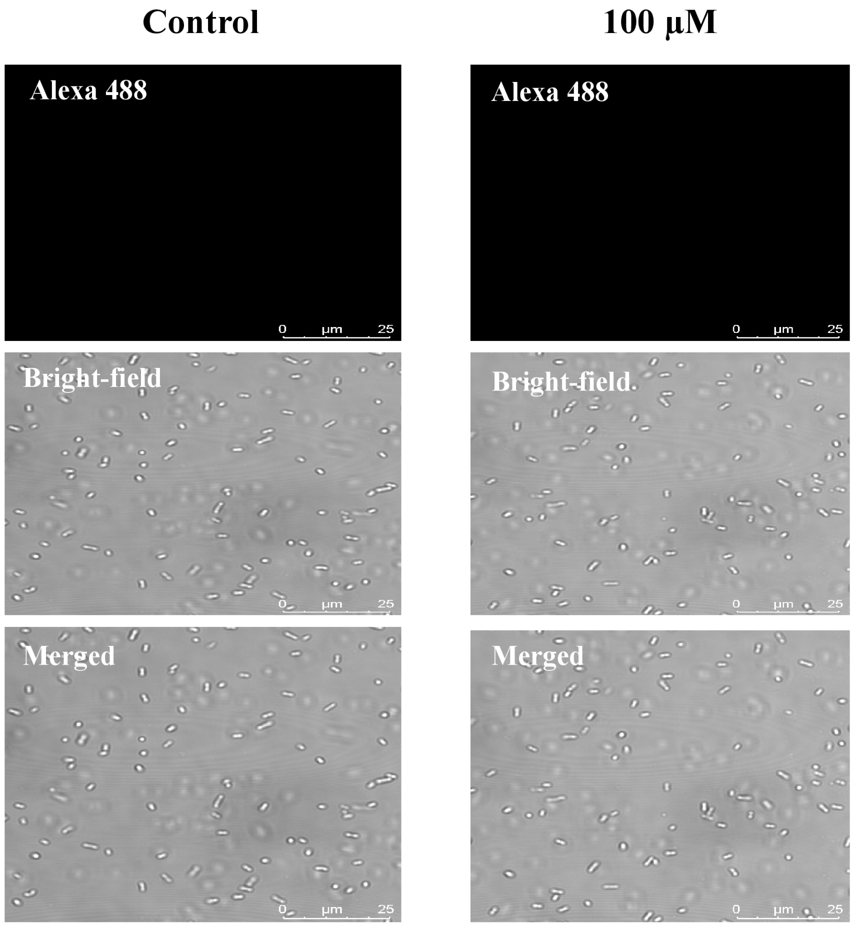

Figure S1: Representative confocal microscopy images visualizing non-binding of IgG-Fc region on *E. coli* surface after incubation with 6His-LPETG peptide (panel 2). The concentration of peptide used is indicated.

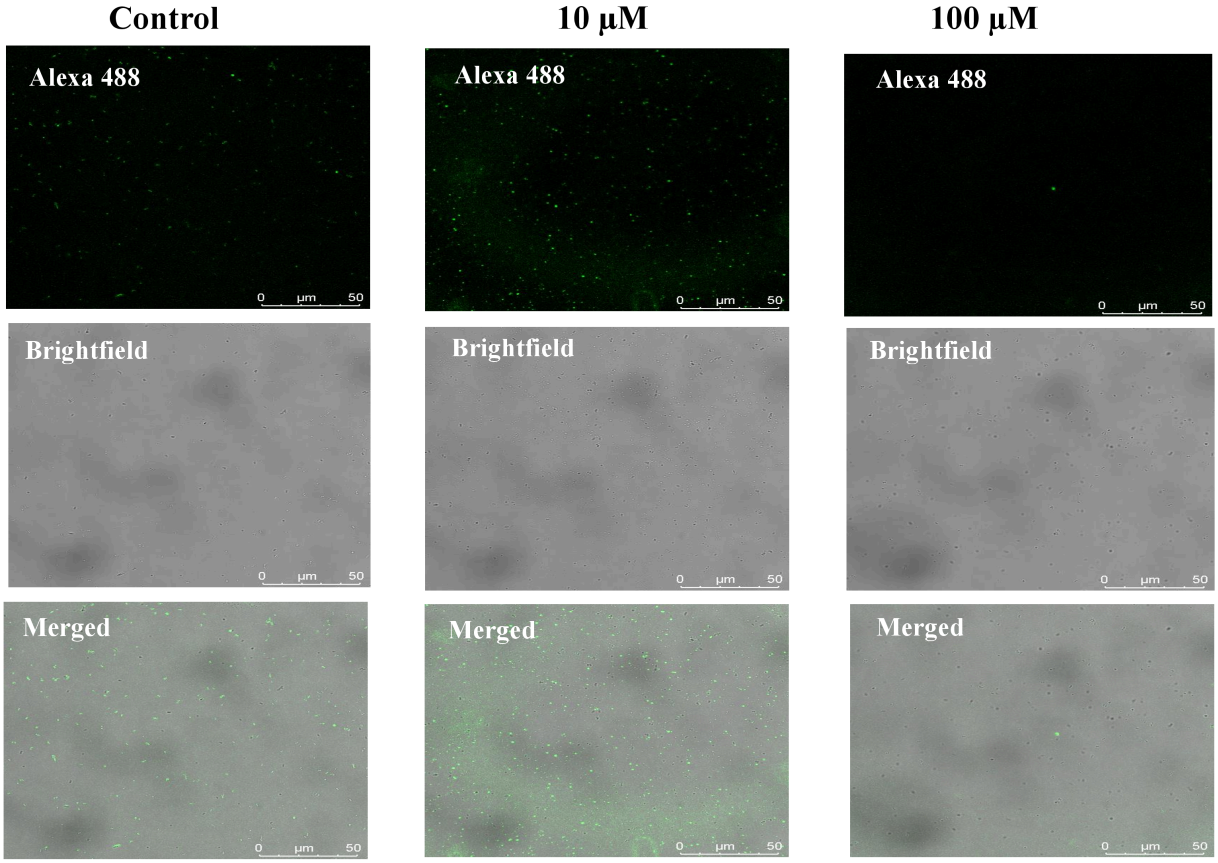

Figure S2: Representative confocal microscopy images visualizing *S. aureus* binding to IgG Fc region after the recruitment of 6His-LPETG peptide on its cell surface (Panel 2 & 3). The concentration of peptide used is indicated.

dPABBs

Design Peptides Against Bacterial Biofilms

OPEN SOURCE DRUG DISCOVERY

Home | Peptide | Protein | Batch | MultiModel | Download | Algorithm | Putative ABPs | Help | Developers | Contact

Go Back

Original Peptide

Peptide Sequence	Mutation Position	SVM score	Prediction	Hydrophobicity	Hydropathicity	Hydrophilicity	Charge	Mol wt
HHHHHHLPETG	No	-0.34	Biofilm-inactive	-0.23	-1.96	-0.20	2.00	1338.59

Mutant Peptides

Peptide Sequence	Mutation Position	SVM score	Prediction	Hydrophobicity	Hydropathicity	Hydrophilicity	Charge	Mol wt
AHHHHHLPETG	1	-0.01	Biofilm-inactive	-0.18	-1.51	-0.20	1.50	1272.52
CHHHHHLPETG	1	-0.13	Biofilm-inactive	-0.19	-1.45	-0.25	1.50	1304.58
DHHHHHLPETG	1	-0.15	Biofilm-inactive	-0.26	-1.99	0.12	0.50	1316.53
EHHHHHLPETG	1	-0.28	Biofilm-inactive	-0.25	-1.99	0.12	0.50	1330.56
FHHHHHLPETG	1	-0.08	Biofilm-inactive	-0.14	-1.42	-0.38	1.50	1348.62
GHHHHHLPETG	1	-0.13	Biofilm-inactive	-0.18	-1.71	-0.15	1.50	1258.50
IHHHHHLPETG	1	-0.01	Biofilm-inactive	-0.13	-1.26	-0.32	1.50	1314.61
KHHHHHLPETG	1	0.00	Biofilm-active	-0.30	-2.03	0.12	2.50	1329.62
LHHHHHLPETG	1	0.04	Biofilm-active	-0.15	-1.33	-0.32	1.50	1314.61
MHHHHHLPETG	1	-0.17	Biofilm-inactive	-0.17	-1.50	-0.27	1.50	1332.64
NHHHHHLPETG	1	-0.14	Biofilm-inactive	-0.26	-1.99	-0.14	1.50	1315.55
PHHHHHLPETG	1	-0.27	Biofilm-inactive	-0.20	-1.82	-0.15	1.50	1298.56
QHHHHHLPETG	1	-0.13	Biofilm-inactive	-0.26	-1.99	-0.14	1.50	1329.58
RHHHHHLPETG	1	-0.07	Biofilm-inactive	-0.36	-2.08	0.12	2.50	1357.63
SHHHHHLPETG	1	-0.06	Biofilm-inactive	-0.22	-1.75	-0.13	1.50	1288.52
THHHHHLPETG	1	-0.20	Biofilm-inactive	-0.21	-1.74	-0.19	1.50	1302.55
VHHHHHLPETG	1	0.02	Biofilm-active	-0.15	-1.29	-0.29	1.50	1300.58
WHHHHHLPETG	1	-0.08	Biofilm-inactive	-0.16	-1.75	-0.46	1.50	1387.66
YHHHHHLPETG	1	-0.13	Biofilm-inactive	-0.20	-1.79	-0.36	1.50	1364.62
HAHHHHLPETG	2	-0.01	Biofilm-inactive	-0.18	-1.51	-0.20	1.50	1272.52
HCHHHHLPETG	2	-0.13	Biofilm-inactive	-0.19	-1.45	-0.25	1.50	1304.58
HDHHHHLPETG	2	-0.15	Biofilm-inactive	-0.26	-1.99	0.12	0.50	1316.53
HEHHHHLPETG	2	-0.28	Biofilm-inactive	-0.25	-1.99	0.12	0.50	1330.56
HFHHHHLPETG	2	-0.08	Biofilm-inactive	-0.14	-1.42	-0.38	1.50	1348.62
HGHHHHLPETG	2	-0.13	Biofilm-inactive	-0.18	-1.71	-0.15	1.50	1258.50
HIHHHHLPETG	2	-0.01	Biofilm-inactive	-0.13	-1.26	-0.32	1.50	1314.61

Developed by: OSDD Unit, CSIR- HQ, New Delhi- 110001

Figure S3. dPABBs database output for prediction of standalone anti-biofilm activity of 6His-LPETG peptide. A negative score signifies that the peptide has no standalone anti-biofilm activity.

dPABBs
Design Peptides Against Bacterial Biofilms

Home | Peptide | Protein | Batch | MultiModel | Download | Algorithm | Putative ABPs | Help | Developers | Contact

Go Back

Original Peptide

Peptide Sequence	Mutation Position	SVM score	Prediction	Hydrophobicity	Hydropathicity	Hydrophilicity	Charge	Mol wt
DYKDDDDKLPETG	No	-1.26	Biofilm-inactive	-0.46	-2.23	1.50	-4.00	1510.70

Mutant Peptides

Peptide Sequence	Mutation Position	SVM score	Prediction	Hydrophobicity	Hydropathicity	Hydrophilicity	Charge	Mol wt
AYKDDDDKLPETG	1	-1.21	Biofilm-inactive	-0.38	-1.82	1.23	-3.00	1466.69
CYKDDDDKLPETG	1	-1.28	Biofilm-inactive	-0.40	-1.77	1.19	-3.00	1498.75
EYKDDDDKLPETG	1	-1.13	Biofilm-inactive	-0.45	-2.23	1.50	-4.00	1524.73
FYKDDDDKLPETG	1	-1.38	Biofilm-inactive	-0.36	-1.75	1.08	-3.00	1542.79
GYKDDDDKLPETG	1	-1.25	Biofilm-inactive	-0.39	-1.99	1.27	-3.00	1452.67
HYKDDDDKLPETG	1	-1.07	Biofilm-inactive	-0.43	-2.21	1.23	-2.50	1532.76
IYKDDDDKLPETG	1	-1.35	Biofilm-inactive	-0.35	-1.62	1.13	-3.00	1508.78
KYKDDDDKLPETG	1	-1.05	Biofilm-inactive	-0.49	-2.26	1.50	-2.00	1523.79
LYKDDDDKLPETG	1	-1.18	Biofilm-inactive	-0.36	-1.67	1.13	-3.00	1508.78
MYKDDDDKLPETG	1	-1.36	Biofilm-inactive	-0.38	-1.82	1.17	-3.00	1526.81
NYKDDDDKLPETG	1	-1.29	Biofilm-inactive	-0.45	-2.23	1.28	-3.00	1509.72
PYKDDDDKLPETG	1	-1.37	Biofilm-inactive	-0.41	-2.08	1.27	-3.00	1492.73
QYKDDDDKLPETG	1	-1.24	Biofilm-inactive	-0.46	-2.23	1.28	-3.00	1523.75
RYKDDDDKLPETG	1	-1.30	Biofilm-inactive	-0.54	-2.31	1.50	-2.00	1551.80
SYKDDDDKLPETG	1	-1.37	Biofilm-inactive	-0.42	-2.02	1.29	-3.00	1482.69
TYKDDDDKLPETG	1	-1.15	Biofilm-inactive	-0.42	-2.02	1.24	-3.00	1496.72
VYKDDDDKLPETG	1	-0.98	Biofilm-inactive	-0.36	-1.64	1.15	-3.00	1494.75
WYKDDDDKLPETG	1	-1.19	Biofilm-inactive	-0.37	-2.03	1.01	-3.00	1581.83
YYKDDDDKLPETG	1	-1.21	Biofilm-inactive	-0.40	-2.06	1.09	-3.00	1558.79
DAKDDDDKLPETG	2	-1.23	Biofilm-inactive	-0.44	-1.99	1.64	-4.00	1418.60
DCKDDDDKLPETG	2	-1.25	Biofilm-inactive	-0.46	-1.94	1.60	-4.00	1450.66
DDKDDDDKLPETG	2	-1.15	Biofilm-inactive	-0.52	-2.40	1.91	-5.00	1462.61
DEKDDDDKLPETG	2	-1.14	Biofilm-inactive	-0.51	-2.40	1.91	-5.00	1476.64
DFKDDDDKLPETG	2	-1.33	Biofilm-inactive	-0.41	-1.92	1.48	-4.00	1494.70
DGKDDDDKLPETG	2	-1.23	Biofilm-inactive	-0.45	-2.16	1.68	-4.00	1404.58

1/1 400

Developed by: OSDD Unit, CSIR- HQ, New Delhi- 110001

Figure S4. dPABBs database output for prediction of standalone anti-biofilm activity of FLAG-LPETG peptide. A negative score signifies that the peptide has no standalone anti-biofilm activity.

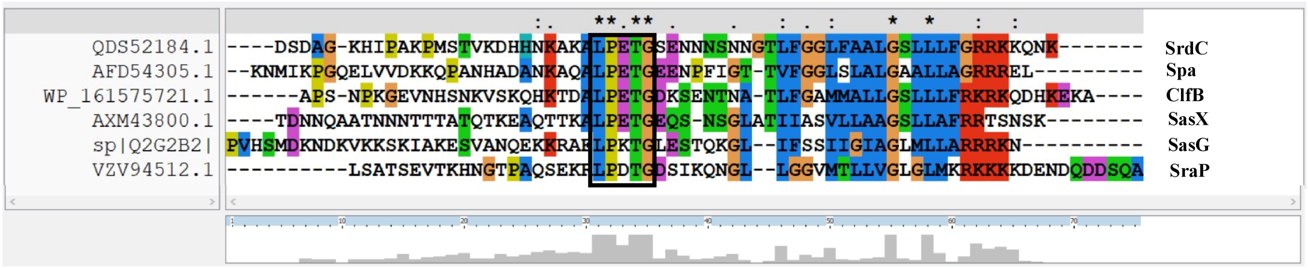

Figure S5: Multiple Sequence Alignment of C-terminal (64 residues) of *S. aureus* proteins (SrdC, Spa, ClfB, SasX, SasG, SraP) involved in biofilm formation using CLUSTALX 2.1. The conserved LPXTG motif is highlighted.
